# Association between composite dietary antioxidant index and coronary heart disease among US adults: a cross-sectional analysis

**DOI:** 10.1186/s12889-023-17373-1

**Published:** 2023-12-05

**Authors:** Ruicong Ma, Xinyang Zhou, Guolin Zhang, Hongying Wu, Yi Lu, Fengyi Liu, Yeting Chang, Yanchun Ding

**Affiliations:** 1 Department of Cardiology, The Second Hospital of Dalian Medical University, No.467 Zhongshan Road, Shahekou District, Dalian, 116021 Liaoning People’s Republic of China; 2 Department of Immunology, College of Basic Medical Science, Dalian Medical University, Dalian, Liaoning 116044 People’s Republic of China

**Keywords:** Coronary heart disease, NHANES, CDAI, Cross-sectional study

## Abstract

**Background:**

The Composite Dietary Antioxidant Index (CDAI) is a dietary antioxidant score that plays a protective role in many diseases, including depression, osteoporosis, papillomavirus infection, etc. However, the association between CDAI and coronary heart disease (CHD) is currently unclear. We aim to explore the correlations between CDAI and the risk of CHD.

**Methods:**

Eligible participants were obtained from the National Health and Nutrition Examination Survey (NHANES) from 1999 to 2018. All participants in this cross-sectional study are required to undergo two separate 24-h dietary recall interviews. Average daily intakes of dietary antioxidants were used to calculate CDAI. CHD status was determined through a questionnaire. Weighted multiple logistic regression models were used to evaluate the relationship between CDAI and CHD. Moreover, we also used restricted cubic spline to explore Non-linear correlations. Sensitivity analysis using unweighted logistic analysis and subgroup analysis were used to demonstrate the stability of the results.

**Results:**

A total of 34,699 participants were eligible for analysis.Compared to the participants without CHD, the participants with CHD showed lower levels of CDAI. After adjusting confounding factors in the multivariate weighted logistic regression model, CDAI was inversely associated with CHD (Q4 vs. Q1, OR = 0.65 (0.51–0.82, *P* < 0.001). Restricted cubic spline showed that there was a negative non-linear correlation (L-shaped) between CDAI and CHD, suggesting a potential saturation effect at higher CDAI levels, with the inflection point of 0.16. Sensitivity analysis showed that the results were stable. No significant statistically interaction was showed in subgroup analysis.

**Conclusions:**

There was a negative non-linear correlation between CDAI and CHD in US adults. However, further prospective studies are still needed to reveal their relationship.

**Supplementary Information:**

The online version contains supplementary material available at 10.1186/s12889-023-17373-1.

## Introduction

Coronary heart disease (CHD) is a chronic inflammatory disease and the leading cause of death worldwide, which accounts for approximately 33% of all deaths globally [1, 2]. CHD has brought large economic burden and medical cost all over the world, especially in the U.S. [3]. Moreover, CHD accounts for one-third of cardiovascular disease costs although the prevention and treatment of CHD has been carried out effectively [4, 5]. CHD can lead to adverse cardiovascular events, such as stroke, heart failure and myocardial infarction [6]. Common risk factors for CHD include hypertension, hyperlipidemia, hyperglycemia and obesity [7–9]. Additionally, some unhealthy lifestyle factors, such as smoking, poor diet and physical inactivity, can promote the progress of atherosclerosis [10–12]. Atherosclerosis is a progressive and chronic condition characterized by the accumulation of fatty deposits, cholesterol, cellular debris, and calcium within the walls of arteries. These deposits form plaques, which can narrow and harden the arteries over time. As a result, the affected arteries become less flexible and have reduced blood flow capacity, eventually leading to CHD [13]. Moreover, if atherosclerosis in the coronary arteries progresses to the point where a plaque ruptures or a blood clot forms at the site of the plaque, it can completely block blood flow to a portion of the heart, causing a heart attack [14]. Preventing CHD is necessary in public health and a healthy diet is the most acceptable way to decrease this burden. The dietary patterns of populations have a substantial impact on the overall burden of CHD. Numerous epidemiological studies have consistently demonstrated that diets high in saturated fats, trans fats, refined sugars, and low in fruits, vegetables, and whole grains are strongly associated with an increased risk of CHD. Conversely, diets characterized by higher consumption of fruits, vegetables, whole grains, lean proteins, and healthy fats have been linked to reduced CHD risk [15, 16]. Furthermore, from a public health perspective, dietary modifications can be implemented across diverse socioeconomic and demographic groups. Changing dietary habits can be achieved through education, policy interventions, and community-based programs.

Oxidative stress and inflammation also play an important role in the development of CHD. Inflammation and oxidative stress are closely related. Inflammation can promote oxidative stress, leading to further inflammation in turn. Therefore, oxidative stress and inflammation can establish a self-sustaining cycle, which can accelerate the development of atherosclerosis [17]. Dietary antioxidants are effective interventions to reduce oxidative stress and inflammation. Antioxidant and anti-inflammatory treatment can limit the progress of atherosclerosis [18, 19]. With the improvement of living standards, people are increasingly concerned about dietary health, especially the antioxidant capacity of food.

The Composite Dietary Antioxidant Index (CDAI) is a composite score composed of multiple dietary antioxidants, including vitamins, zinc, selenium and carotenoid [20, 21]. CDAI can reflect an individual’s antioxidant profile. CDAI was devised in accordance with the demonstrable anti-inflammatory efficacy of dietary antioxidants, predicated upon their capacity to mitigate pro-inflammatory factors such as tumor necrosis factor-α and interleukin-1β. The antioxidant components used to calculate CDAI primarily include vitamin A, vitamin C, vitamin E, β-carotene, selenium, and zinc [22]. These compounds are considered to possess significant antioxidant activity. Researchers can employ CDAI to investigate the association between dietary antioxidant capacity and chronic diseases such as cardiovascular diseases, cancer, diabetes, among others [23–25]. This contributes to a better understanding of the potential role of diet in disease prevention and management. Furthermore, CDAI can also be utilized to provide dietary recommendations for individuals, aiming to enhance their antioxidant protective capacity and improve their overall health status [26].

CDAI is closely related to many diseases, including cancer, depression, osteoporosis, papillomavirus infection [27–30]. However, the association between CDAI and CHD remains unclear. Therefore, our study aimed to investigate the link between CDAI and CHD risk, which may provide assistance for the prevention and therapy of CHD.

## Materials and methods

### Data source and study participates

**Fig. 1:**
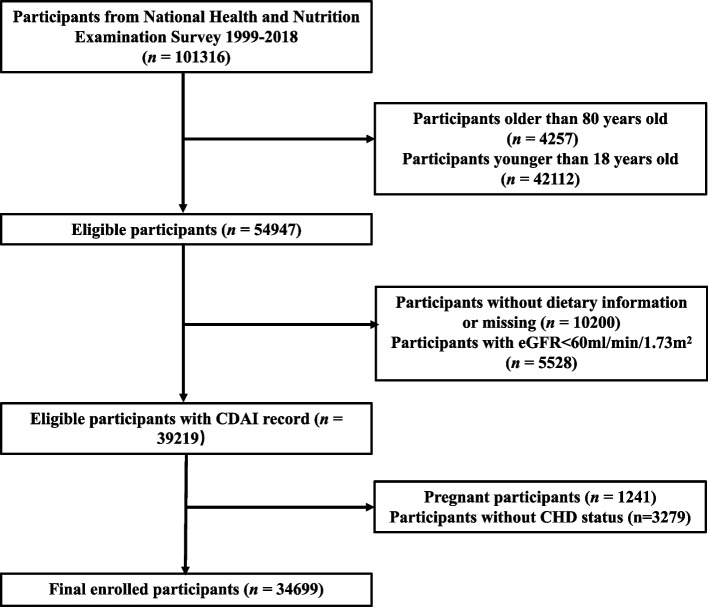
The flow chart of participant selection

Data from the National Health and Nutrition Examination Survey (NHANES) database, which encompasses 85 various topics, including smoking, alcohol consumption, weight, dietary intake, physical health, and activity (www.cdc.gov/nchs/nhanes.com), was utilized in our study. We used this data to assess the health and nutritional status of adults aged 18–80 in the United States. In the present study, we excluded participants aged over 80 years, they typically have different lifestyles and health conditions, often presenting with long-term chronic illnesses and medication treatments, which could potentially interfere with the study's results. The data samples were collected across various states and counties in the United States. From all NHANES participants between 1999 and 2018 (*n* = 101,316), we excluded individuals whom older than 80 years (*n* = 4,257) or younger than 18 years (*n* = 42,112), those without dietary data or with incomplete dietary data (having only one day of dietary record as opposed to two days, and the participants without dietary interview data, *n* = 10,200), the individuals with an estimated glomerular filtration rate (eGFR) of < 60 ml/min/1.73 m^2^ (*n* = 5,528), pregnant participants (*n* = 1,241) and participants without CHD status (with no response to the CHD status in the questionnaire) (*n* = 3,279). Finally, a total of 34,699 participants were included in the analytical sample. The details of the screening process and the size of the participants are shown in Fig. 1.

### CDAI

CDAI data were derived from the dietary recall survey of NHANES participants. All participants are required to undergo two separate 24-h dietary recall interviews. The first dietary recall was conducted in person. The second dietary interview was proceeded 3–10 days by telephone. Average daily intakes can be calculated based on 2 days recall data. CDAI is composed of six dietary antioxidants, including zinc, selenium, carotenoids, vitamin A, C and E. The measurement method proposed by Wright was used to calculate CDAI [20]. We subtracted the universal mean and divided the results by its worldwide standard deviation from the six dietary minerals and vitamins as follows:

$$\mathrm{CDAI}={\sum }_{i=1}^{n=6}\frac{individual\ Intake-Mean}{SD}$$

### Diagnosis of CHD

Based on previous NHANES research, CHD is mainly diagnosed according to a history of CHD [31]. Participants were asked “whether a doctor or other health professional has ever told you that you had CHD?”, and persons who answered “yes” were considered to have CHD.

### Covariates

In this study, we selected several covariates related to CHD based on previously published research.

We extracted demographic data from the NHANES database, which encompassed information pertaining to age (in years), gender (categorized as male or female), racial/ethnic background (including white, black, Mexican, other Hispanic, and other categories), and educational attainment (categorized as less than high school, high school, and post-high school education). This information was derived from the NHANES demographic questionnaire. Body mass index (BMI) was calculated by dividing weight [kg] by the square of height [m^2^]. According to the data based on the questionnaire collected by NHANES, we obtained smoking status (yes/no) and alcohol consumption (yes/no). From laboratory examination data, we collected cardiovascular and metabolic measurements, including fasting blood glucose (FBG) (mmol/L), glycated hemoglobin (HbA1c) (%), triglycerides (TG) (mmol/L), total cholesterol (TC) (mmol/L), high-density lipoprotein cholesterol (HDL-C) (mmol/L), low-density lipoprotein cholesterol (LDL-C) (mmol/L) and creatinine. We calculated the estimated glomerular filtration rate (eGFR) based on the creatinine data of participants provided by NHANES using the Chronic Kidney Disease Epidemiology Collaboration (CKD-EPI) method [32]. Hypertension was diagnosed based on guidelines provided by the Joint National Committee on Prevention, Detection, Evaluation, and Treatment of High Blood Pressure. We applied hypertension evaluation criteria: SBP ≥ 140 mmHg or DBP ≥ 90 mmHg and the patients using antihypertensive medications for the period of being investigated. We applied diabetes evaluation criteria: doctor diagnosis as diabetes, HbA1c ≥ 6.5%, fasting glucose ≥ 7.0 mmol/L, random blood glucose ≥ 11.1 mmol/L, 2 h OGTT blood glucose ≥ 11.1 mmol/L, or being treated with diabetes drugs and insulin.

### Statistical analysis

This study used a series of statistical methods to analyze the data. Initially, we divided the data into two groups: CHD and non-CHD. Continuous variables were presented as means (95% confidence intervals (CI)), and categorical variables were presented as proportions (95% CI). We used independent sample t-tests (for continuous variables) and chi-square tests (for categorical variables) to compare differences between the two groups, with *P* values < 0.05 considered statistically significant. Next, we conducted a correlation analysis of CDAI. We calculated each component's mean (95% CI) and compared the differences between CHD and non-CHD groups. To compare cardiovascular and metabolic indicators between different CDAI quartiles, we used a one-way analysis of variance (ANOVA). Thirdly, we performed weighted logistic regression analyses to investigate the association between CDAI and CHD. We constructed three models: unadjusted, Model I, and Model II. Model I was adjusted for age, sex, and race. Model II was adjusted for age, sex, race, education levels, smoking, drinking, BMI, hypertension, DM, SBP, DBP, TG, TC, LDL-C, HDL-C and eGFR. Results were presented as odds ratios (ORs) with 95% CIs. We used restricted cubic spline to explore Non-linear correlations. Energy adjustment was unnecessary because CDAI incorporates relatively limited components of antioxidant food items that have a significant impact on energy supply. Numerous studies have already analyzed public data from the NHANES database to investigate risk factors for various diseases. Among these studies, some researchers have utilized weighted analysis methods, while others have employed unweighted approaches. Although NHANES employs complex sampling techniques to enhance the representativeness and applicability of findings, the conclusions derived from weighted and unweighted analyses can sometimes diverge. In this study, we conducted sensitivity analysis by unweighted logistic regression analysis. Regarding the choice of employing an unweighted analysis method for sensitivity analysis, we offer the following explanation. Numerous studies have already analyzed public data from the NHANES database to investigate risk factors for various diseases. Among these studies, some researchers have utilized weighted analysis methods, while others have employed unweighted approaches. Although NHANES employs complex sampling techniques to enhance the representativeness and applicability of findings, the conclusions derived from weighted and unweighted analyses can sometimes diverge. Therefore, we also carried out unweighted logistic regression analysis to reaffirm our results. Moreover, subgroup analysis was used to further validate the stability of results.

## Results

### The baseline characteristics of participants

A total of 34,699 screened participants were involved, of which 1,009 were diagnosed with CHD. Table 1 shows the baseline characteristics of all participants, including age, sex, race, BMI, smoking, alcohol consumption, education level, SBP, DBP, hypertension, diabetes, fasting blood glucose, glycated hemoglobin, eGFR, TG, TC, LDL-C and HDL-C.

**Table 1:** Clinical characteristics of study population

Variables	Overall (*n* = 34699)	Non-CHD (*n* = 33694)	CHD (*n* = 1005)	*P* value
**Age, %**				< 0.001***
18–39 years	39.07 (37.51, 40.63)	40.00 (38.94, 41.05)	3.71 (2.24, 5.18)	
40–59 years	41.51 (39.51, 43.51)	41.78 (40.94, 42.63)	31.01 (26.80, 35.22)	
> 60 years	19.42 (18.31, 20.53)	18.22 (17.50, 18.94)	65.28 (61.10, 69.47)	
**Gender, %**				< 0.001***
Female	50.34 (48.27, 52.41)	50.89 (50.32, 51.47)	29.23 (25.56, 32.90)	
Male	49.66 (47.75, 51.57)	49.11 (48.53, 49.68)	70.77 (67.10, 74.44)	
**Race/ethnicity, %**				< 0.001***
White	68.28 (63.78, 72.77)	67.98 (65.77, 70.18)	79.74 (76.48, 83.01)	
Black	10.58 (9.64, 11.53)	10.70 (9.56, 11.85)	6.02 (4.69, 7.36)	
Mexican	8.63 (7.59, 9.68)	8.75 (7.59, 9.92)	4.17 (2.85, 5.49)	
Other Hispanic	5.63 (4.84, 6.42)	5.69 (4.87, 6.52)	3.14 (1.86, 4.41)	
Others	6.88 (6.30, 7.45)	6.87 (6.26, 7.49)	6.93 (4.84, 9.01)	
**Education levels, %**				< 0.001***
Less than high school	5.07 (4.66, 5.49)	5.00 (4.58, 5.42)	7.89 (6.06, 9.72)	
High school or equivalent	34.18 (32.30, 36.05)	34.04 (32.81, 35.28)	40.03 (35.51, 44.55)	
College or above	60.70 (57.95, 63.44)	60.95 (59.55, 62.36)	52.08 (47.39, 56.76)	
**BMI, kg/m**^**2**^**, %**				< 0.001***
Normal weight	30.33 (28.94, 31.71)	30.89 (30.01, 31.77)	18.45 (15.33, 21.56)	
Obesity	35.97 (34.29, 37.65)	35.96 (35.03, 36.88)	48.27 (43.62, 52.91)	
Over weight	32.88 (31.37, 34.39)	33.15 (32.38, 33.92)	33.29 (29.88, 36.70)	
**Smoking, %**				0.95
No	77.48 (74.40, 80.56)	77.50 (76.66, 78.35)	77.63 (73.87, 81.38)	
Yes	22.49 (21.30, 23.68)	22.50 (21.65, 23.34)	22.37 (18.62, 26.13)	
**Drinking, %**				0.07
No	9.62 (8.70, 10.53)	10.32 (9.39, 11.25)	8.10 (5.98, 10.22)	
Yes	84.08 (80.59, 87.57)	89.68 (88.75, 90.61)	91.90 (89.78, 94.02)	
**DM, %**				< 0.001***
No	88.49 (85.01, 91.97)	89.17 (88.72, 89.63)	62.46 (58.77, 66.16)	
Yes	11.51 (10.91, 12.11)	10.83 (10.37, 11.28)	37.54 (33.84, 41.23)	
**FBG, mg/dl**	5.82 (5.79, 5.86)	5.79 (5.76, 5.83)	6.93 (6.62, 7.23)	< 0.001***
**HbA1c, %**	5.56 (5.54, 5.57)	5.54 (5.53, 5.56)	6.13 (6.04, 6.22)	< 0.001***
**Hypertension, %**				< 0.001***
No	65.87 (63.20, 68.53)	66.89 (66.07, 67.70)	26.94 (23.52, 30.36)	
Yes	34.13 (32.60, 35.67)	33.11 (32.30, 33.93)	73.06 (69.64, 76.48)	
**SBP, mmHg**	120.98 (120.65, 121.31)	120.82 (120.49, 121.15)	127.01 (125.57, 128.45)	< 0.001***
**DBP, mmHg**	71.67 (71.35, 71.99)	71.74 (71.42, 72.06)	69.10 (67.98, 70.22)	< 0.001***
**eGFR, mL/min/1.73 m**^**2**^	97.74 (97.27, 98.20)	98.11 (97.64, 98.58)	83.42 (82.44, 84.40)	< 0.001***
**TG, mmol/L**	1.48 (1.45, 1.51)	1.47 (1.44, 1.50)	1.72 (1.58, 1.85)	< 0.001***
**TC, mmol/L**	5.07 (5.05, 5.09)	5.08 (5.06, 5.11)	4.66 (4.55, 4.78)	< 0.001***
**LDL-C, mmol/L**	3.00 (2.98, 3.02)	3.01 (2.99, 3.03)	2.53 (2.45, 2.62)	< 0.001***
**HDL-C, mmol/L**	1.37 (1.37, 1.38)	1.38 (1.37, 1.39)	1.24 (1.20, 1.27)	< 0.001***
**Angina, %**				< 0.001***
No	98.07 (94.34, 101.81)	99.04 (98.89, 99.20)	65.30 (61.31, 69.29)	
Yes	1.80 (1.58, 2.03)	0.96 (0.80, 1.11)	34.70 (30.71, 38.69)	
**Congestive heart failure, %**				< 0.001***
No	98.50 (94.74, 102.25)	99.11 (99.00, 99.22)	77.54 (74.10, 80.98)	
Yes	1.44 (1.27, 1.60)	0.89 (0.78, 1.00)	22.46 (19.02, 25.90)	
**Heart attack, %**				< 0.001***
No	97.43 (93.71, 101.14)	98.74 (98.59, 98.89)	49.50 (46.00, 53.00)	
Yes	2.51 (2.26, 2.77)	1.26 (1.11, 1.41)	50.50 (47.00, 54.00)	

Continuous data were presented as the mean and 95% confidence interval, category data were presented as the proportion and 95% confidence interval

*CHD* coronary heart disease, *BMI* body mass index, *DM* diabetes, *FBG* fast blood glucose, *HbA1c* glycosylated hemoglobin, *SBP* systolic blood pressure, *DBP* diastolic blood pressure, *eGFR* estimated glomerular filtration rate, *TG* triglycerides, *TC* total cholesterol, *LDL-C* low-density lipoprotein cholesterol, *HDL-C* high-density lipoprotein cholesterol

^***^ *P* value < 0.001, ** *P* value < 0.01, * *P* value < 0.05

Table 1 shows significant differences in clinical characteristics between the CHD group and non-CHD group. The patients in the CHD group were older (*P* < 0.001), more male (*P* < 0.001), and the race was also statistically different (*P* < 0.001). Compared with the non-CHD group, patients with CHD have lower education level. Moreover, the proportion of obesity, DM, hypertension, angina, congestive heart failure and heart attack are higher in the CHD group. We also found differences between cardiovascular and metabolic measurements, such as FBG, HbA1c, TG, TC, LDL-C and HDL-C (*P* < 0.001). In addition, the eGFR in patients with CHD were significantly lower than those without CHD (*P* < 0.001). Additionally, the baseline characteristics of participants across different quartiles of the CDAI are shown in Table S1. Variables were categorized into four quartiles of the CDAI to investigate the effect of varying diet quality on these variables. Among these variables, significant differences were found for age, sex, race, smoking, alcohol consumption, education level, hypertension, DM, angina, congestive heart failure, heart attack, BMI, LDL-C and HDL-C between CDAI within four quartiles. To understand the relationship between CDAI and the risk of CHD, we compared the CDAI components between CHD and non-CHD groups (Table 2). Overall, the CDAI was significantly higher in the non-CHD group than in the CHD group (0.51, 95% CI: 0.43–0.59 vs. 0.32, 95% CI: 0.23–0.41, *P* < 0.001***). It is worth noting that in the CDAI components, the non-CHD group had significantly higher Vitamin A, Zinc and Selenium than the CHD group. We compared the cardiovascular metabolic indicators between the Quartile of CDAI. Table 3 shows that among various cardiovascular metabolic indicators, HbA1c, TG and LDL-C show significant differences between the four Quartile of CDAI.

**Table 2:** CDAI and its components among non-CHD group and CHD group

Variables	Overall (*n* = 34699)	Without CHD (*n* = 33694)	CHD (*n* = 1005)	*P* value
CDAI	0.44 (0.37, 0.52)	0.51 (0.43, 0.59)	0.32 (0.23, 0.41)	< 0.001***
Vitamin A, mcg	584.17 (574.47, 593.88)	588.97 (577.34, 600.61)	574.91 (563.12, 586.71)	0.047*
Vitamin C, mg	78.91 (77.30, 80.51)	79.34 (77.46, 81.21)	78.08 (76.03, 80.12)	0.28
Vitamin E, mg	7.95 (7.84, 8.07)	8.01 (7.87, 8.14)	7.85 (7.70, 7.99)	0.06
Zinc, mg	11.54 (11.43, 11.65)	11.65 (11.52, 11.77)	11.34 (11.17, 11.50)	< 0.001***
Selenium, mcg	112.67 (111.73, 113.61)	113.58 (112.47, 114.70)	110.91 (109.54, 112.29)	0.002**
Carotenoid, mcg	8946.11 (8744.33, 9147.90)	8938.80 (8713.24, 9164.36)	8960.23 (8682.10, 9238.36)	0.89

Data were presented as the mean and 95% confidence interval

*CHD* coronary heart disease, *CDAI* composite dietary antioxidant index

^***^ *P* value < 0.001, ** *P* value < 0.01, * *P* value < 0.05

**Table 3:** Comparison of cardiac metabolism parameters of the study population grouped by CDAI quartiles

Variables	Overall	CDAI-Q1	CDAI-Q2	CDAI-Q3	CDAI-Q4	*P* value
FBG, mmol/L	5.82 (5.79, 5.86)	5.85 (5.79, 5.91)	5.80 (5.75, 5.85)	5.85 (5.78, 5.93)	5.80 (5.74, 5.85)	0.4
HbA1c, %	5.56 (5.54, 5.57)	5.58 (5.55, 5.61)	5.57 (5.54, 5.59)	5.56 (5.54, 5.59)	5.52 (5.49, 5.55)	0.01*
TC, mmol/L	5.07 (5.05, 5.09)	5.10 (5.07, 5.14)	5.08 (5.05, 5.11)	5.07 (5.04, 5.10)	5.05 (5.01, 5.08)	0.08
TG, mmol/L	1.37 (1.37, 1.38)	1.36 (1.35, 1.37)	1.36 (1.35, 1.37)	1.37 (1.36, 1.39)	1.39 (1.38, 1.41)	0.004**
LDL-C, mmol/L	3.00 (2.98, 3.02)	3.03 (2.98, 3.07)	3.05 (3.01, 3.08)	2.97 (2.94, 3.00)	2.96 (2.92, 3.00)	< 0.001***
HDL-C, mmol/L	1.48 (1.45, 1.51)	1.49 (1.43, 1.54)	1.48 (1.44, 1.53)	1.48 (1.42, 1.54)	1.47 (1.41, 1.52)	0.94

Data were presented as the mean and 95% confidence interval

*CDAI* composite dietary antioxidant index, *FBG* fast blood glucose, *HbA1c* glycosylated hemoglobin, *TC* total cholesterol, *TG* triglycerides, *LDL-C* low-density lipoprotein cholesterol, *HDL-C* high-density lipoprotein cholesterol

^***^ *P* value < 0.001, ** *P* value < 0.01, * *P* value < 0.05

### Association between CDAI and CHD

As a continuous variable, a negative correlation was showed between CDAI and CHD, with an OR of 0.94 (95%CI: 0.92–0.96) in unadjusted logistic regression analysis (Table 4). In Model 3, CDAI remained significantly negatively correlated with CHD (OR = 0.95, 95%CI: 0.92–0.97). Compared with the Q1 quartile of CDAI, the Q4 quartile has a lower risk of CHD prevalence in Model 2 (0R = 0.65, 95%CI: 0.51–0.82).

**Table 4:** Weighted logistic regression analysis on the association between CDAI and CHD

	Non-adjusted model		Model I		Model II	
	**OR [95% CI]**	***P* value**	**OR [95% CI]**	***P* value**	**OR [95% CI]**	***P* value**
Continuous CDAI	0.94 (0.92,0.96)	< 0.001***	0.93 (0.91,0.96)	< 0.001***	0.95 (0.92,0.97)	< 0.001***
CDAI-Q1	Reference	-	Reference	-	Reference	-
CDAI-Q2	0.93 (0.73,1.19)	0.55	0.83 (0.65,1.08)	0.16	0.89 (0.69,1.15)	0.37
CDAI-Q3	0.71 (0.57,0.88)	0.002**	0.63 (0.50,0.79)	< 0.001***	0.67 (0.53,0.84)	< 0.001***
CDAI-Q4	0.59 (0.47,0.75)	< 0.001***	0.58 (0.46,0.73)	< 0.001***	0.65 (0.51,0.82)	< 0.001***

Data are presented as OR (95% CI). Model I adjusted for age, sex, and race/ethnicity. Model II adjusted for age, sex, race, education levels, smoking, drinking, BMI, hypertension, DM, SBP, DBP, TG, TC, LDL-C, HDL-C, and eGFR

*BMI* body mass index, *DM* diabetes, *TG* triglycerides, *TC* total cholesterol, *LDL-C* low-density lipoprotein cholesterol, *HDL-C* high-density lipoprotein cholesterol, *eGFR* estimated glomerular filtration rate

^***^ *P* value < 0.001, ** *P* value < 0.01, * *P* value < 0.05

### Restricted cubic spline

**Fig. 2:**
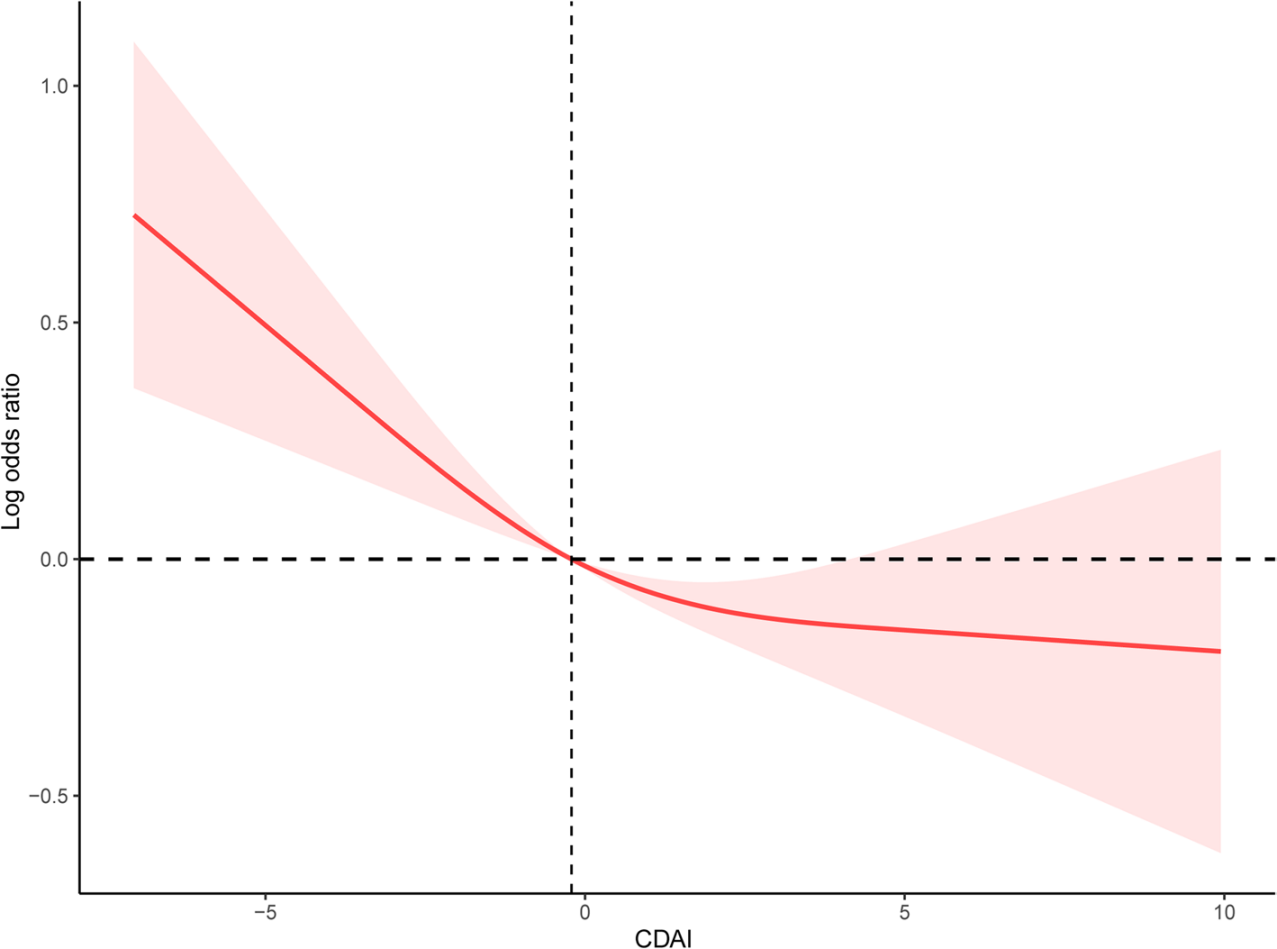
The correlation between CDAI and the risk of CHD

We used restricted cubic spline to analyze the correlation between CDAI and CHD. The results indicated that there was a non-linear negative correlation between CDAI and the risk of CHD. Furthermore, the risk of CHD was more significantly reduced before the median. As CDAI scores increased, the odds of CHD exhibited a gradual decline up to a certain threshold. Beyond the inflection point (0.16), the protective effect seemed to stabilize, suggesting a potential saturation effect at higher CDAI levels (Fig. 2).

### Sensitivity analysis

Similarly, sensitivity analysis adopting unweighted logistic analysis indicated that the Q4 quartile has a lower risk of CHD prevalence in Model 2 (0R = 0.66, 95%CI: 0.53–0.81) compared with the Q1 quartile of CDAI (Table 5). The results represents that the negative association between CDAI and CHD was stable.

**Table 5:** Unweighted logistic regression analysis on the association between CDAI and CHD in sensitivity analysis

	Non-adjusted model		Model I		Model II	
	**OR [95% CI]**	***P* value**	**OR [95% CI]**	***P* value**	**OR [95% CI]**	***P* value**
Continuous CDAI	0.94 (0.92,0.96)	< 0.001***	0.95 (0.93,0.97)	< 0.001***	0.96 (0.93,0.98)	< 0.001***
CDAI-Q1	Reference	-	Reference	-	Reference	-
CDAI-Q2	0.83 (0.70,0.98)	0.03*	0.78 (0.66,0.92)	0.002**	0.84 (0.70,1.00)	0.048*
CDAI-Q3	0.72 (0.60,0.85)	0.002**	0.69 (0.59,0.82)	< 0.001***	0.76 (0.62,0.92)	0.004**
CDAI-Q4	0.56 (0.47,0.67)	< 0.001***	0.61 (0.51,0.73)	< 0.001***	0.66 (0.53,0.81)	< 0.001***

Data are presented as OR (95% CI). Model I adjusted for age, sex, and race/ethnicity. Model II adjusted for age, sex, race, education levels, smoking, drinking, BMI, hypertension, DM, SBP, DBP, TG, TC, LDL-C, HDL-C, and eGFR

*BMI* body mass index, *DM* diabetes, *TG* triglycerides, *TC* total cholesterol, *LDL-C* low-density lipoprotein cholesterol, *HDL-C* high-density lipoprotein cholesterol, *eGFR* estimated glomerular filtration rate

^***^ *P* value < 0.001, ** *P* value < 0.01, * *P* value < 0.05

### Subgroups analysis

**Fig. 3:**
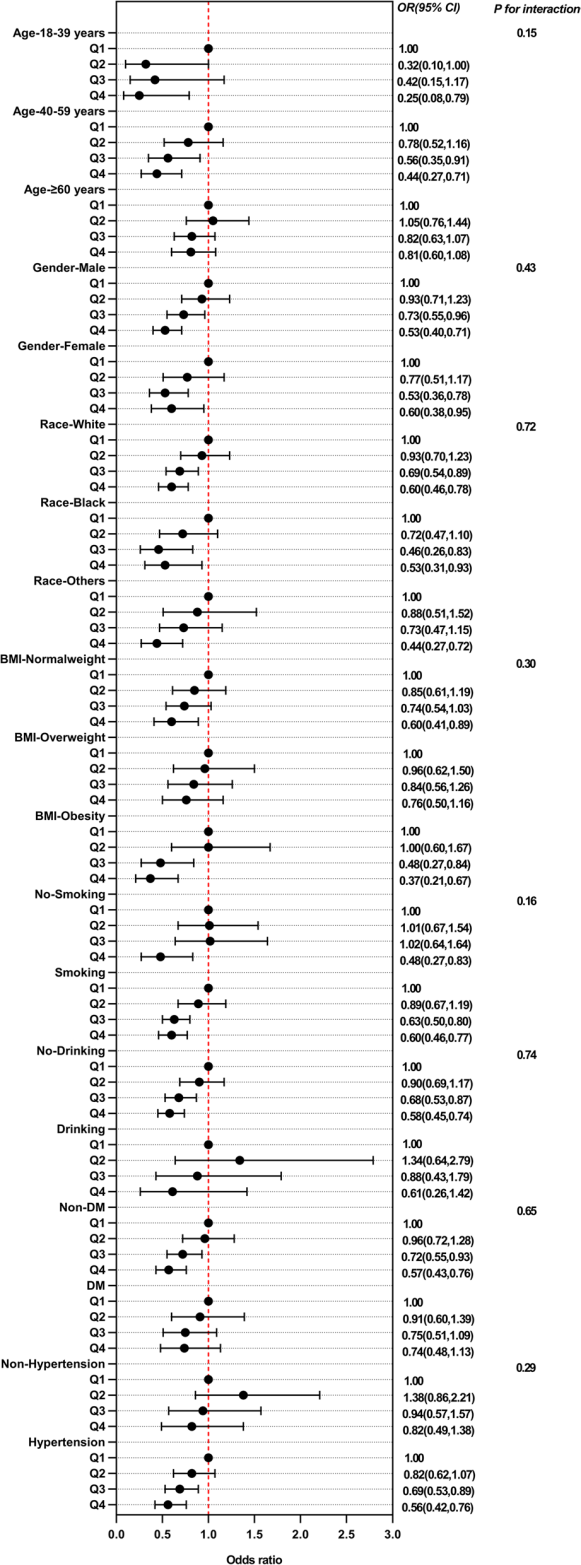
Subgroup analysis of multi-variable adjusted association of CDAI with the risk of CHD

Subgroup analysis was used to explore the potential relationship between CDAI and CHD in different subgroups based on age, gender, BMI, smoking, drinking, hypertension and DM (Fig. 3, Table S2). Trend testing suggests statistical differences in CDAI among women, obesity, and smoking subgroups. However, there was no difference between the other subgroups of the impact of CDAI on CHD.

## Discussion

Dietary antioxidants play a protective role in many diseases. In this study, we found that there was a negative correlation between CDAI and CHD in US adults after adjusting for confounding factors. Fitted smoothing curves indicate the Non-linear negative correlation. Trend testing suggests statistical differences in CDAI among women, obesity, and smoking subgroups, however, there was no difference between the other subgroups of the impact of CDAI onCHD. Poor dietary habits can lead to chronic inflammation, which plays a major role in many metabolic diseases [33]. A pro-inflammatory diet can amplify the inflammatory response in the body by increasing oxidative stress and immune disorders. Unhealthy food habits are usually related to higher levels of inflammatory cytokines, which can promote the development of atherosclerosis [34]. Pro-inflammatory habits can lead to the production of reactive oxygen species (ROS), which causes endothelial dysfunction and chronic vascular inflammation [35]. CDAI was developed according to their effective anti-inflammatory effect based on pro-inflammatory factors, reflecting an individual’s antioxidant profile. Previous studies have found that CDAI is associated with the risk of all-cause mortality among US adults, and in a linear pattern. Eating a diet rich in antioxidants can significantly prevent mortality among the general population in the United States [36]. Another study confirmed that low antioxidant diet is associated with increased colorectal cancer risk [37]. Besides, another cross-sectional study revealed a negative nonlinear association between CDAI and depression, and the he inflection point is 0.16. Before the inflection point, for the increase of each unit in CDAI, the risk of depression decreases by 30%. After the inflection point, it was found that for each additional unit, the risk of depression decreased by 11% [38]. Additionally, improving the intake of dietary antioxidants also plays a protective role in many other diseases, including cancer, depression, osteoporosis, papillomavirus infection [39].

In this study, our results based on 34,699 participants revealed significant increases in CDAI levels of adults with CHD. Previous studies have shown that inflammation and oxidative stress lead to the damage of endothelial and smooth muscle cells, which promote the development of CHD [40, 41]. We found that the non-CHD group had significantly higher Vitamin A, Zinc and Selenium, which ameliorate oxidative stress and progression of inflammation. Therefore, patients with CHD have lower levels of CDAI, which is related to the intake of more antioxidant foods. Recent studies have found that dietary antioxidant capacity is negatively correlated with CHD and biomarkers of oxidative stress [42, 43]. It is widely recognized that antioxidant food can reduce ROS by using molecules to exhibit antioxidant activities and regulate gene expression. In other words, antioxidants are cellular signaling regulators [44]. Zinc and selenium are important antioxidants against oxidative stress. A nested case–control study based on 1,621 incident CHD cases and 1,621 non-CHD cases showed that incident CHD was inversely associated with selenium [45]. Selenium binds to selenoproteins to inhibit lipid peroxidation and cell damage from oxidative stress [46]. Another study performed multiple logistic regression on blood measurements of 3,541 participants, including 1,253 patients diagnosed with CHD and 2288 healthy participants. The results indicated that low blood zinc concentration is an independent risk factor for CHD [47]. NHANES is conducted in two-year cycles, and each cycle includes a representative sample of the U.S. population. These cycles are designed to provide nationally representative estimates and encompass a range of demographic groups, ensuring a robust and diverse dataset. Given that our study aimed to explore the association between dietary antioxidant index and coronary heart disease within a representative sample of the U.S. adult population, we chose to incorporate data from multiple cycles to maximize the sample size and diversity. This approach allows for a more comprehensive analysis, encompassing a broader spectrum of age groups, geographic regions, and socio-economic backgrounds, which enhances the generalizability of our findings. Moreover, coronary heart disease is a complex and multifactorial condition that develops over time, often influenced by long-term dietary habits. By including data from multiple cycles, we were able to capture a more extensive timeframe of dietary exposure, which can be valuable in understanding potential associations between diet and disease. In summary, the wide time period of data collection in our cross-sectional study is a deliberate choice based on the NHANES dataset's structure and our research objectives. It enhances the robustness and generalizability of our findings, allowing us to explore the relationship between dietary antioxidant index and coronary heart disease within a diverse and representative sample of U.S. adults. We believe this approach contributes to the richness and depth of our analysis. Of note, zinc and selenium are two important components of CDAI. Previous studies are consistent with our research findings that patients with CHD have lower CDAI, especially in zinc and selenium. Our results may provide assistance for the prevention and therapy of CHD.

Interestingly, The trend testing found that the negative correlation was particularly significant among female. We considered that this phenomenon resulted from different responses of men and women to foodborne inflammation and oxidative stress [48–50].

This study has clarified for the first time that CDAI is related to the risk of CHD. It may have certain guiding significance for health management among US adults. The study is based on the National Health and Nutrition Examination Survey (NHANES), which is a nationally representative dataset. This enhances the generalizability of the findings to the broader U.S. adult population. NHANES typically includes a large and diverse sample size, allowing for a robust analysis and increasing the statistical power to detect associations. While cross-sectional studies have limitations, they can provide valuable insights into associations between variables. In this case, it allows for a snapshot of the relationship between CDAI and coronary heart disease among U.S. adults. The study focuses on a composite dietary antioxidant index (CDAI), which provides a comprehensive measure of antioxidant intake from various dietary sources. This approach may better capture the overall dietary antioxidant status compared to studying individual antioxidants. However, we want to mention some limitations of our study. First, we cannot confirm a causal relationship between CDAI and CHD considering the type of cross-sectional study. Second, CHD was diagnosed according to a history of CHD which may lead to subjective bias. Thirdly, due to significant differences in dietary habits among different races, the conclusions from the study mainly apply to the American population and further research is needed to explore the association between CDAI and CHD. Furthermore, there is the possibility that not all patients may accurately know the type of their heart diseases considering the definition of the outcome was ascertained by questionnaire.

## Conclusion

Our study shows that there is a negative non-linear correlation between CDAI and CHD in US adults. However, further prospective studies are still needed to reveal their relationship.

### Supplementary Information

**Additional file 1:** **Table S1.** Clinical characteristics of study population grouped by CDAI quartiles.

**Additional file 2:** **Table S2.** Subgroup analysis of multi-variable adjusted association of CDAI with the risk of CHD.

## Data Availability

The datasets generated during the current study are available in database (https://www.cdc.gov/nchs/nhanes/).
