# Supplementary material for: Association between composite dietary antioxidant index and coronary heart disease among US adults: a cross-sectional analysis
Source: BMC Public Health. 2023 Dec 5;23:2426. doi: 10.1186/s12889-023-17373-1 (PMC10699074; doi:10.1186/s12889-023-17373-1)
Supplement: Supplementary file 1 — Additional file 1: Table S1. Clinical characteristics of study population grouped by CDAI quartiles. [file 12889_2023_17373_MOESM1_ESM.docx]

**Table 1. Clinical characteristics of study population grouped by CDAI quartiles.**

| Variables | Overall | CDAI-Q1 | CDAI-Q2 | CDAI-Q3 | CDAI-Q4 | *P* value |
| --- | --- | --- | --- | --- | --- | --- |
| Age, % |  |  |  |  |  | <0.001*** |
| 18-39 years | 19.42 [18.31, 20.53] | 20.00 [18.88, 21.13] | 20.72 [19.46, 21.98] | 19.89 [18.71, 21.06] | 17.33 [15.89, 18.78] |  |
| 40–59 years | 39.07 [37.51, 40.63] | 39.85 [38.28, 41.43] | 38.55 [37.26, 39.83] | 38.18 [36.39, 39.97] | 39.76 [38.19, 41.32] |  |
| >60 years | 41.51 [39.51, 43.51] | 40.14 [38.66, 41.63] | 40.73 [39.41, 42.06] | 41.93 [40.44, 43.42] | 42.91 [41.43, 44.40] |  |
| Gender, % |  |  |  |  |  | <0.001*** |
| Female | 50.34 [48.27, 52.41] | 56.20 [55.10, 57.30] | 50.05 [48.78, 51.33] | 48.75 [47.39, 50.10] | 47.35 [46.03, 48.67] |  |
| Male | 49.66 [47.75, 51.57] | 43.80 [42.70, 44.90] | 49.95 [48.67, 51.22] | 51.25 [49.90, 52.61] | 52.65 [51.33, 53.97] |  |
| Race/ethnicity, % |  |  |  |  |  | <0.001*** |
| White | 68.28 [63.78, 72.77] | 63.53 [60.57, 66.48] | 68.19 [65.72, 70.66] | 70.28 [68.06, 72.49] | 70.30 [68.12, 72.48] |  |
| Black | 10.58 [9.64, 11.53] | 14.42 [12.72, 16.12] | 10.47 [9.24, 11.70] | 9.12 [8.02, 10.22] | 8.97 [7.95, 10.00] |  |
| Mexican | 8.63 [7.59, 9.68] | 8.46 [7.16, 9.76] | 8.81 [7.54, 10.08] | 8.58 [7.39, 9.77] | 8.67 [7.39, 9.96] |  |
| Other Hispanic | 5.63 [4.84, 6.42] | 6.61 [5.36, 7.86] | 5.65 [4.68, 6.61] | 5.31 [4.48, 6.15] | 5.11 [4.37, 5.85] |  |
| Others | 6.88 [6.30, 7.45] | 6.98 [6.16, 7.81] | 6.88 [6.08, 7.69] | 6.71 [5.94, 7.47] | 6.95 [6.16, 7.73] |  |
| Education levels, % |  |  |  |  |  | <0.001*** |
| Less than high school | 5.07 [4.66, 5.49] | 7.37 [6.58, 8.16] | 5.31 [4.66, 5.96] | 4.41 [3.93, 4.88] | 3.64 [3.13, 4.15] |  |
| High school or equivalent | 34.18 [32.30, 36.05] | 43.10 [41.47, 44.73] | 35.48 [33.73, 37.22] | 30.91 [29.31, 32.52] | 28.95 [27.38, 30.52] |  |
| College or above | 60.70 [57.95, 63.44] | 49.53 [47.85, 51.22] | 59.21 [57.31, 61.12] | 64.68 [62.96, 66.41] | 67.41 [65.65, 69.17] |  |
| BMI, % |  |  |  |  |  | 0.001** |
| Normal weight | 30.33 [28.94, 31.71] | 30.34 [29.14, 31.54] | 29.14 [27.85, 30.44] | 30.01 [28.67, 31.36] | 32.59 [30.91, 34.28] |  |
| Obesity | 35.97 [34.29, 37.65] | 37.69 [36.28, 39.09] | 37.36 [35.74, 38.98] | 35.39 [33.78, 37.00] | 35.00 [33.38, 36.62] |  |
| Over weight | 32.88 [31.37, 34.39] | 31.97 [30.52, 33.42] | 33.50 [32.12, 34.88] | 34.60 [33.18, 36.02] | 32.41 [31.11, 33.71] |  |
| Smoking, % |  |  |  |  |  | <0.001*** |
| No | 77.48 [74.40, 80.56] | 68.31 [66.82, 69.80] | 77.02 [75.68, 78.36] | 80.56 [79.39, 81.73] | 82.50 [81.34, 83.65] |  |
| Yes | 22.49 [21.30, 23.68] | 31.69 [30.20, 33.18] | 22.98 [21.64, 24.32] | 19.44 [18.27, 20.61] | 17.50 [16.35, 18.66] |  |
| Drinking, % |  |  |  |  |  | <0.001*** |
| No | 9.62 [8.70, 10.53] | 12.40 [11.34, 13.46] | 10.32 [9.27, 11.37] | 10.34 [8.97, 11.72] | 8.40 [7.34, 9.46] |  |
| Yes | 84.08 [80.59, 87.57] | 87.60 [86.54, 88.66] | 89.68 [88.63, 90.73] | 89.66 [88.28, 91.03] | 91.60 [90.54, 92.66] |  |
| DM, % |  |  |  |  |  | 0.001** |
| No | 88.49 [85.01, 91.97] | 87.34 [86.31, 88.36] | 88.09 [87.32, 88.86] | 88.50 [87.64, 89.37] | 89.78 [88.95, 90.60] |  |
| Yes | 11.51 [10.91, 12.11] | 12.66 [11.64, 13.69] | 11.91 [11.14, 12.68] | 11.50 [10.63, 12.36] | 10.22 [9.40, 11.05] |  |
| FBG, mg/dl | 5.82 [5.79, 5.86] | 5.85 [5.79, 5.91] | 5.80 [5.75, 5.85] | 5.85 [5.78, 5.93] | 5.80 [5.74, 5.85] | 0.4 |
| HbA1c, % | 5.56 [5.54, 5.57] | 5.58 [5.55, 5.61] | 5.57 [5.54, 5.59] | 5.56 [5.54, 5.59] | 5.52 [5.49, 5.55] | 0.01* |
| Hypertension, % |  |  |  |  |  | 0.004** |
| No | 65.87 [63.20, 68.53] | 64.21 [62.79, 65.64] | 64.88 [63.50, 66.27] | 66.79 [65.42, 68.17] | 67.20 [65.74, 68.66] |  |
| Yes | 34.13 [32.60, 35.67] | 35.79 [34.36, 37.21] | 35.12 [33.73, 36.50] | 33.21 [31.83, 34.58] | 32.80 [31.34, 34.26] |  |
| SBP, mmHg | 120.98 [120.65, 121.31] | 121.74 [121.22, 122.26] | 121.27 [120.81, 121.72] | 120.79 [120.28, 121.30] | 120.28 [119.82, 120.75] | <0.001*** |
| DBP, mmHg | 71.67 [71.35, 71.99] | 71.45 [71.04, 71.86] | 71.62 [71.20, 72.05] | 71.59 [71.19, 71.99] | 71.97 [71.58, 72.37] | 0.11 |
| eGFR, mL/min/1.73 m^2^ | 97.74 [97.27, 98.20] | 98.09 [97.46, 98.73] | 97.59 [96.99, 98.19] | 97.50 [96.86, 98.13] | 97.81 [97.13, 98.48] | 0.29 |
| Triglycerides, mmol/L | 1.48 [1.45, 1.51] | 1.49 [1.43, 1.54] | 1.48 [1.44, 1.53] | 1.48 [1.42, 1.54] | 1.47 [1.41, 1.52] | 0.94 |
| Total cholesterol, mmol/L | 5.07 [5.05, 5.09] | 5.10 [5.07, 5.14] | 5.08 [5.05, 5.11] | 5.07 [5.04, 5.10] | 5.05 [5.01, 5.08] | 0.08 |
| LDL-C, mmol/L | 3.00 [2.98, 3.02] | 3.03 [2.98, 3.07] | 3.05 [3.01, 3.08] | 2.97 [2.94, 3.00] | 2.96 [2.92, 3.00] | <0.001*** |
| HDL-C, mmol/L | 1.37 [1.37, 1.38] | 1.36 [1.35, 1.37] | 1.36 [1.35, 1.37] | 1.37 [1.36, 1.39] | 1.39 [1.38, 1.41] | 0.004** |
| Angina, % |  |  |  |  |  | 0.01* |
| No | 98.07 [94.34, 101.81] | 97.60 [97.09, 98.11] | 98.20 [97.90, 98.51] | 98.34 [97.94, 98.74] | 98.53 [98.20, 98.86] |  |
| Yes | 1.80 [1.58, 2.03] | 2.40 [1.89, 2.91] | 1.80 [1.49, 2.10] | 1.66 [1.26, 2.06] | 1.47 [1.14, 1.80] |  |
| Congestive heart failure, % |  |  |  |  |  | <0.001*** |
| No | 98.50 [94.74, 102.25] | 97.85 [97.47, 98.22] | 98.47 [98.17, 98.78] | 98.81 [98.56, 99.06] | 98.99 [98.78, 99.20] |  |
| Yes | 1.44 [1.27, 1.60] | 2.15 [1.78, 2.53] | 1.53 [1.22, 1.83] | 1.19 [0.94, 1.44] | 1.01 [0.80, 1.22] |  |
| Heart attack, % |  |  |  |  |  | <0.001*** |
| No | 97.43 [93.71, 101.14] | 96.58 [96.03, 97.13] | 97.28 [96.86, 97.70] | 97.64 [97.20, 98.08] | 98.26 [97.94, 98.58] |  |
| Yes | 2.51 [2.26, 2.77] | 3.42 [2.87, 3.97] | 2.72 [2.30, 3.14] | 2.36 [1.92, 2.80] | 1.74 [1.42, 2.06] |  |

Continuous data were presented as the mean and 95% confidence interval, category data were presented as the proportion and 95% confidence interval. CDAI, composite dietary antioxidant index; CHD, coronary heart disease; BMI, body mass index; DM, diabetes; FBG, fast blood glucose; HbA1c, glycosylated hemoglobin; SBP, systolic blood pressure; DBP, diastolic blood pressure; eGFR, estimated glomerular filtration rate; LDL-C, low-density lipoprotein cholesterol; HDL-C, high-density lipoprotein cholesterol. *** *P* value<0.001, ** *P* value<0.01, * *P* value<0.05.
