# Supplementary material for: Association between composite dietary antioxidant index and coronary heart disease among US adults: a cross-sectional analysis
Source: BMC Public Health. 2023 Dec 5;23:2426. doi: 10.1186/s12889-023-17373-1 (PMC10699074; doi:10.1186/s12889-023-17373-1)
Supplement: Supplementary file 2 — Additional file 2: Table S2. Subgroup analysis of multi-variable adjusted association of CDAI with the risk of CHD. [file 12889_2023_17373_MOESM2_ESM.docx]

**Table S2: Subgroup analysis of multi-variable adjusted association of CDAI with the risk of CHD.**

| Variable name | Q1 | Q2 | Q3 | Q4 | p for trend | p for interaction |
| --- | --- | --- | --- | --- | --- | --- |
|  |  |  |  |  |  | 0.15 |
| Age 18-39 years | ref | 0.32(0.10,1.00) | 0.42(0.15,1.17) | 0.25(0.08,0.79) | 0.09 |  |
| Age 40-59 years | ref | 0.78(0.52,1.16) | 0.56(0.35,0.91) | 0.44(0.27,0.71) | 0.6 |  |
| Age≥60 years | ref | 1.05(0.76,1.44) | 0.82(0.63,1.07) | 0.81(0.60,1.08) | 0.19 |  |
|  |  |  |  |  |  | 0.43 |
| Gender-Male | ref | 0.93(0.71,1.23) | 0.73(0.55,0.96) | 0.53(0.40,0.71) | 0.5 |  |
| Gender-Female | ref | 0.77(0.51,1.17) | 0.53(0.36,0.78) | 0.60(0.38,0.95) | 0.01 |  |
|  |  |  |  |  |  | 0.72 |
| Race-White | ref | 0.93(0.70,1.23) | 0.69(0.54,0.89) | 0.60(0.46,0.78) | 0.13 |  |
| Race-Black | ref | 0.72(0.47,1.10) | 0.46(0.26,0.83) | 0.53(0.31,0.93) | 0.27 |  |
| Race-Others | ref | 0.88(0.51,1.52) | 0.73(0.47,1.15) | 0.44(0.27,0.72) | 1 |  |
|  |  |  |  |  |  | 0.3 |
| BMI-Normalweight | ref | 0.85(0.61,1.19) | 0.74(0.54,1.03) | 0.60(0.41,0.89) | 0.26 |  |
| BMI-Overweight | ref | 0.96(0.62,1.50) | 0.84(0.56,1.26) | 0.76(0.50,1.16) | 0.67 |  |
| BMI-Obesity | ref | 1.00(0.60,1.67) | 0.48(0.27,0.84) | 0.37(0.21,0.67) | <0.001 |  |
|  |  |  |  |  |  | 0.16 |
| No-Smoking | ref | 1.01(0.67,1.54) | 1.02(0.64,1.64) | 0.48(0.27,0.83) | 0.56 |  |
| Smoking | ref | 0.89(0.67,1.19) | 0.63(0.50,0.80) | 0.60(0.46,0.77) | 0.02 |  |
|  |  |  |  |  |  | 0.74 |
| No-Drinking | ref | 0.90(0.69,1.17) | 0.68(0.53,0.87) | 0.58(0.45,0.74) | 0.09 |  |
| Drinking | ref | 1.34(0.64,2.79) | 0.88(0.43,1.79) | 0.61(0.26,1.42) | 0.19 |  |
|  |  |  |  |  |  | 0.65 |
| Non-DM | ref | 0.96(0.72,1.28) | 0.72(0.55,0.93) | 0.57(0.43,0.76) | 0.28 |  |
| DM | ref | 0.91(0.60,1.39) | 0.75(0.51,1.09) | 0.74(0.48,1.13) | 0.87 |  |
|  |  |  |  |  |  | 0.29 |
| Non-Hypertension | ref | 1.38(0.86,2.21) | 0.94(0.57,1.57) | 0.82(0.49,1.38) | 0.62 |  |
| Hypertension | ref | 0.82(0.62,1.07) | 0.69(0.53,0.89) | 0.56(0.42,0.76) | 0.45 |  |

Continuous data were presented as the mean and 95% confidence interval, category data were presented as the proportion and 95% confidence interval. BMI, body mass index; DM, diabetes.
